# Evaluating a Structured Self‐Management Kit for Type 2 Diabetes: A Mixed Methods Service Evaluation in Primary Care

**DOI:** 10.1111/jep.70724

**Published:** 2026-11-04

**Authors:** Nitish Nachiappan, David A. Scott, Mark Stone, Nachiappan Chockalingam

**Affiliations:** ^1^ University College London London UK; ^2^ University of Southampton Southampton UK; ^3^ Staffordshire and Stoke on Trent ICB Staffordshire UK; ^4^ Reading Central PCN Reading UK; ^5^ Staffordshire University Stoke‐on‐Trent UK

**Keywords:** assistive devices, diabetic foot, health economics, orthotics, self‐management, service evaluation

## Abstract

**Rationale, Aims and Objectives:**

Type 2 diabetes places increasing pressure on health services, and engagement with structured education and routine preventative care remains variable. Practical self‐management tools may help people translate advice into daily routines. This service evaluation examined the implementation, acceptability and early outcomes of the MyHealthKits Diabetic Wellness Kit in routine primary care.

**Method:**

A prospective convergent mixed methods service evaluation was conducted across 13 general practices within three Primary Care Networks in Staffordshire and Stoke‐on‐Trent. Adults with type 2 diabetes received a Diabetic Wellness Kit containing foot care tools, educational materials and access to online resources. Health‐related quality of life and healthcare use were measured at baseline and after 12 weeks using EQ‐5D‐5L and MODRUM questionnaires. Quantitative data were analysed descriptively, with paired EQ‐5D utility change assessed using a paired *t*‐test. Semi‐structured interviews with a purposive sample explored usability and perceived impact and were analysed thematically.

**Results:**

A total of 782 kits were distributed. Of these, 131 participants completed both baseline and follow‐up questionnaires; three were excluded because they reported not receiving the kit, leaving 128 in the primary analysis cohort. Mean EQ‐ 5D utility scores increased from 0.61 to 0.70, with 65% of complete responders reporting improvement. Self‐reported healthcare use was lower across all service categories, with an estimated mean cost difference of £288 per participant. Sensitivity analyses excluding inpatient episodes, and excluding both inpatient episodes and day case appointments, gave estimated mean cost differences of £186 and £183 respectively. Qualitative findings indicated improved self‐efficacy, daily care routines, confidence and independence.

**Conclusion:**

The kit was feasible, acceptable and associated with early improvements in reported wellbeing and self‐management. Findings should be interpreted cautiously because the evaluation had no control group, high attrition, short follow‐up, and self‐reported healthcare use. Further controlled studies with longer follow‐up and verified healthcare use data are needed.

## 1 Introduction

The global rise in chronic disease, particularly type 2 diabetes mellitus (T2DM), has placed increasing demands on health systems, with the UK seeing a sustained rise in both prevalence and associated complications. Nationally, an estimated 3.96 million people are living with T2DM, accounting for approximately 90% of all diabetes cases [1]. Within Staffordshire and Stoke on Trent, approximately 79,400 people were recorded on GP diabetes registers in 2022/23, representing 8.25% of the registered population aged 17 years and over. Type 2 diabetes accounts for the majority of diabetes cases in the region. [2, 3, 4]. Also, this growing burden of diabetes is not evenly distributed. In SSoT, men make up over 56% of recorded T2DM cases, and individuals from minority ethnic backgrounds now account for over 9%, an increasing share over the past 5 years [4]. Notably, most people with T2DM in the region fall within the 40–79 age range, a population at higher risk of developing serious complications such as neuropathy, ulcers, and amputation. Despite the known benefits of early intervention and education, only 14% of newly diagnosed individuals in SSoT attended a structured diabetes education programme in 2021 [4]. This low uptake is further compounded by wider inequalities in access to routine care. For instance, annual foot checks dropped to just 46% during the pandemic recovery period and remain suboptimal at 78% in 2022–2023. Completion of all eight National Institute for Health and Care Excellence (NICE) recommended care processes reached only 55.7% [4, 5, 6].

The consequences of these care gaps are significant. Up to one in three people with diabetes will develop a foot ulcer during their lifetime, and recurrence is common. Approximately 40% of individuals experience a recurrence within 1 year of healing, and 65% within 5 years [7]. The cost to the NHS is substantial. Around 90% of expenditure related to diabetic foot complications is spent on ulceration, with unhealed ulcers and amputations costing up to £8786 and £16,941 respectively in the first year of care [7]. In SSoT, 150 diabetes related lower limb amputations between 2017 and 2023 cost the system over £2.4 million, with the highest rates concentrated in the most deprived areas [4]. Addressing this burden requires scalable, cost‐effective solutions that empower people to self‐manage their condition.

A range of self‐management interventions have been developed to support people living with type 2 diabetes, including structured education programmes, digital health technologies, personalised care planning, and behaviour change interventions [8, 9, 10]. While many of these approaches have demonstrated benefits, participation and sustained engagement remain challenging, particularly among people living in areas of greater social and economic deprivation. NICE recommends structured education for adults with type 2 diabetes, and programmes such as DESMOND have been evaluated in routine care settings [11]. Digital health interventions and personalised care planning have also shown promise in supporting self‐management, although engagement and sustained participation remain variable. Existing interventions often rely on the provision of information or require ongoing interaction with healthcare professionals and digital platforms. In contrast, there is relatively little evidence exploring whether practical assistive products can help individuals translate self‐care advice into daily routines and behaviours. The use of simple, low‐cost tools that support foot care, self‐monitoring, and independent management represents a potentially scalable approach to addressing this gap.

The *MyHealthKits* Diabetic Wellness Kit was developed in response to this need. As reported by Nachiappan et al. [12] and Kulkarni et al. [13], these kits include tools such as a foot mirror, magnifying glass, moisturiser, foot file, bamboo socks, and educational resources tailored to support foot health and general diabetes management. Early evaluations indicate that recipients not only use these items regularly but also report increased confidence, improved daily routines, and in some cases, reduced need for GP visits. Importantly, the design of these kits aligns with broader theoretical frameworks for behaviour change [8]. Drawing on self‐determination theory, the kits aim to foster autonomy, competence, and relatedness, which are key psychological drivers of long‐term self‐care adherence [13]. Participants described personalising their use of the kit, integrating checks into daily life, and involving family members in care practices.

Building on previous evaluations, the *MyHealthKits* Diabetic Wellness Kit was implemented to support people living with type 2 diabetes in managing their condition at home. This service evaluation aimed to examine patterns of kit use, acceptability, patient‐reported outcomes and self‐reported healthcare utilisation over 12 weeks. The findings are intended to inform future decisions on how practical assistive products might be embedded into routine care pathways as a complementary approach to self‐management support.

## 2 Methods

### 2.1 Study Design and Setting

This was a prospective service evaluation conducted within the Staffordshire and Stoke‐on‐Trent Integrated Care System. This evaluation used a convergent mixed methods design. Quantitative questionnaire data and qualitative interview data were collected during the same evaluation period and analysed separately before being brought together during interpretation. The quantitative component explored changes in health‐related quality of life and self‐reported healthcare use, while the qualitative component explored participant experiences, usability, and perceived impact. The qualitative findings were used to help explain how participants used the kit and how it may have supported daily self‐management routines. The aim was to assess the implementation, usage patterns, patient‐reported outcomes and potential cost implications of the Diabetic Wellness Kit, an intervention designed to support self‐management in people with type 2 diabetes mellitus (T2DM). The kit was developed through a process of co‐production involving clinicians and patients based in Staffordshire and has previously been evaluated in community settings within Stoke‐on‐Trent [12, 13]. This project was conducted as a service evaluation of an existing NHS‐supported intervention and was reviewed using the Health Research Authority, UK decision tool, which confirmed that NHS research ethics approval was not required. No clinical procedures or changes to treatment were introduced as part of this evaluation. All participants provided written informed consent before taking part in the questionnaire and interview components. Data were collected and stored in accordance with NHS information governance requirements.

### 2.2 Participants and Recruitment

The intervention was implemented across 13 general practices within three Primary Care Networks (PCNs). Recruitment commenced on 15 July 2024, initially targeting 10 practices across two PCNs to enable comparative analysis. Following lower than anticipated uptake, a planned expansion was undertaken in August 2024 to include a third PCN and three additional practices. Eligible participants were adults with a confirmed diagnosis of T2DM. Recruitment strategies included visual posters placed in participating practices and direct text message invitations sent to individuals identified via GP‐held diabetes registers. All patients who registered for the programme were sent a kit. Those who consented were invited to participate in the evaluation. Written informed consent was obtained from all evaluation participants.

### 2.3 Intervention

The Diabetic Wellness Kit was developed to promote diabetic foot care and general self‐management, in line with NICE guidelines. Each kit contained a telescopic inspection mirror with light, magnifying glass, foot file, seamless diabetic‐friendly socks, medical‐grade insoles, pedometer, measuring tape, and an exercise cloth to encourage physical activity. Moisturiser was also included to support regular skin care, particularly for the feet. The kit was designed as a practical home‐based self‐management intervention rather than a clinical treatment. Participants were advised to use the items as part of their usual daily self‐care routines, particularly for foot inspection, skin care, mobility support and physical activity. Use of individual kit components was not monitored continuously. Instead, participant use and perceived usefulness of the items were explored through follow‐up questionnaires and semi‐structured interviews. Formal implementation fidelity assessment was not undertaken, as the evaluation was designed to examine real‐world uptake and experience rather than controlled delivery under trial conditions. In addition to the physical items, participants received printed educational materials on foot health and diabetes self‐care. They were also provided with access to an online portal containing electronic resources, including lifestyle advice and diabetic‐friendly recipes. Kits were assembled centrally and delivered directly to participants' homes following registration. Participants received written instructions on how to use the kit items safely as part of usual self‐care. The kit was not intended to replace routine diabetes care, clinical foot assessment, or contact with healthcare professionals. Participants were advised to seek clinical advice from their GP practice, podiatry service, or usual diabetes care team if they noticed a new foot wound, broken skin, increasing redness, swelling, discharge, pain, signs of infection, or any other concerning change. No additional clinical foot risk assessment was undertaken as part of this service evaluation.

### 2.4 Data Collection

Data collection was conducted using a secure online evaluation portal. Two validated instruments were used: EQ‐5D‐5L, to assess health‐related quality of life, and the Modular Resource‐Use Measure (MODRUM) [14, 15], to assess medication adherence and healthcare resource utilisation. Participants were asked to recall healthcare service use related to their diabetes over the 12 weeks prior to receiving the kit and the 12 weeks following its use. This included consultations with healthcare professionals and hospital admissions, as captured through the MODRUM questionnaire. In addition to quantitative measures, a purposive sample of participants was invited to take part in semi‐structured telephone interviews between December 2024 and March 2025. All interview participants provided informed consent. The interview guide explored experiences of receiving and using the kit, perceived usefulness of individual items, changes in self‐care routines, confidence, independence and suggestions for improvement. Interview notes and transcripts were reviewed by members of the evaluation team. Coding was undertaken using an inductive thematic approach, and emerging themes were discussed within the team. Disagreements in coding or interpretation were resolved through discussion. Clinical data, including HbA1c, diabetes duration, comorbidities, medication history and foot risk status, were not extracted from GP records.

### 2.5 Data Analysis

As this was a service evaluation, the analysis was primarily descriptive and was intended to characterise patterns of change rather than formally test hypotheses. Descriptive analysis was conducted on data from participants who completed both baseline and follow‐up questionnaires. Health outcomes were expressed as EQ‐5D utility values, where 1 represents full health and 0 represents death [16]. EQ‐5D 5 L responses were mapped to EQ‐5D 3 L values using the published crosswalk algorithm [17] to allow use of the established UK EQ‐ 5D 3 L value set [18]. As mapped values may differ from directly valued EQ‐5D‐5L utilities, the results should be interpreted as estimates of health‐related quality of life rather than definitive utility gains. Paired changes in EQ‐5D utility scores between baseline and follow‐up were assessed using a paired *t*‐test. Mean change, standard error and 95% confidence intervals were calculated for participants with paired baseline and follow‐up data.

Self‐reported healthcare resource utilisation data were analysed across both 12‐week periods (pre‐ and post‐intervention). Unit costs were applied using nationally published NHS sources [19, 20]. Individual‐level costs were calculated by multiplying reported resource use by the corresponding unit cost. The economic component was an exploratory cost consequence and resource use analysis based on self‐reported healthcare utilisation. It was not designed as a formal cost‐effectiveness analysis. The estimated cost of each kit was £50, including product acquisition, assembly and delivery. Programme administration and evaluation costs were not included.

## 3 Results

### 3.1 Recruitment and Response Rates

A total of 859 patients engaged with the Diabetic Wellness Kit programme across the participating sites. Of these, 782 individuals received a kit delivered directly to their homes. At baseline, 740 participants submitted EQ‐5D‐5L and MODRUM questionnaires, comprising 396 complete and 344 partial responses. After data cleaning, 286 responses were retained for analysis. At follow‐up, 424 responses were received (190 complete, 234 partial), of which 156 were usable.

Baseline characteristics of participants included in the primary analysis cohort and baseline‐only responders are presented in Table 1.

**TABLE 1:** Baseline characteristics of complete responders and baseline‐only responders.

Characteristic	Complete responders *n* = 128	Baseline‐only responders *n* = 158
Age, years, mean (SD); median (IQR)	59.9 (11.8); 62.5 (55.0 to 68.0)	54.9 (10.9); 55.0 (48.0 to 62.0)
Female, n (%)	65 (50.8)	76 (48.1)
Male, n (%)	63 (49.2)	81 (51.3)
BMI, kg/m^2^, mean (SD); median (IQR)	32.9 (7.5); 31.2 (27.7 to 36.6)	33.4 (8.2); 31.7 (27.7 to 37.3)

Compared with baseline‐only responders, complete responders were older on average, while sex distribution and BMI were broadly similar. These data provide context for interpreting the findings and assessing the generalisability of the evaluation. Detailed clinical characteristics, including HbA1c, medication history, diabetes duration and comorbidity status, were not available because these data were not extracted from clinical records as part of the service evaluation.

### 3.2 Quantitative Findings

Of the 782 participants who received a Diabetic Wellness Kit, 131 completed both baseline and follow‐up questionnaires. Three were excluded because they reported not receiving the kit, leaving 128 participants in the primary analysis cohort. These participants formed the cohort for evaluating changes in health‐related quality of life and healthcare resource utilisation over the 12 weeks before and after receiving the kit. Participant flow through the evaluation is shown in Figure 1, including the number of individuals who engaged with the programme, received a kit, submitted questionnaires, were retained after data cleaning, and were included in the primary analysis.

**FIGURE 1:**
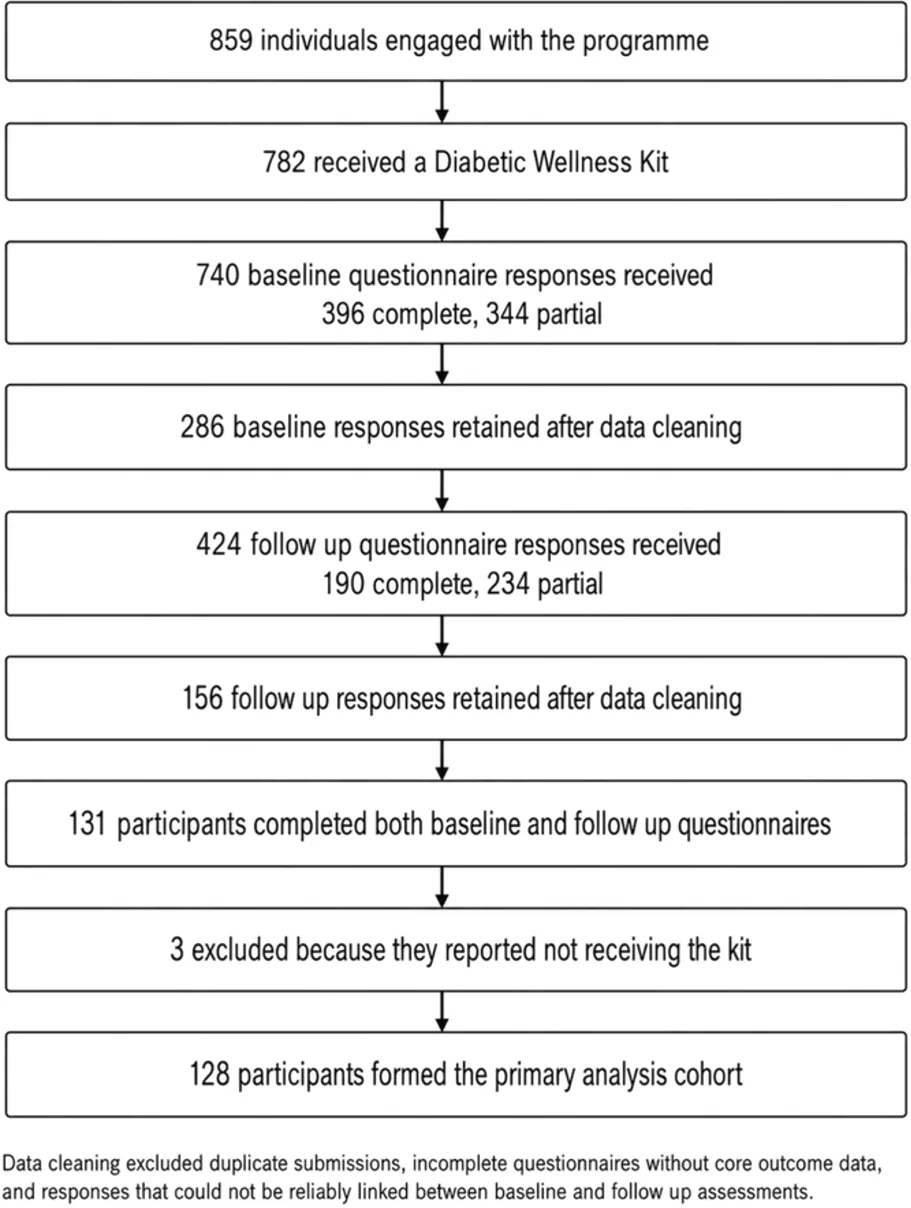
Participant flow through the Diabetic Wellness Kit evaluation.

Mean utility scores, derived from EQ‐5D‐5L responses and converted using the published EQ‐5D‐3L mapping algorithm, increased from 0.61 (SD 0.281) at baseline to 0.70 (SD 0.322) at follow‐up, among participants in the primary analysis cohort. The mean paired difference was 0.082 (95% CI 0.014, 0.151), and this change was statistically significant using a paired *t*‐test (*p* = 0.021). Sixty‐five percent of complete responders showed an improvement in utility score over the 12‐week period following receipt of the kit. No minimally important difference was specified in advance, so this finding should be interpreted as an observed increase in health‐related quality of life among complete responders, rather than evidence of clinical effectiveness. The most commonly used healthcare resources during the evaluation period were outpatient appointments, GP consultations, and nurse or podiatrist visits. In contrast, inpatient admissions, day case attendances, and home visits were infrequently reported. Across all service categories, a reduction in healthcare utilisation was observed in the post‐intervention period compared to baseline (Table 2).

**TABLE 2:** Healthcare resource usage in previous 12 weeks (complete responders).

	At baseline		At 12‐week follow‐up		
Healthcare service	Total	Average per patient	Total	Average per patient	Difference (Follow‐up minus baseline)
Accident and Emergency visits	33	0.26	22	0.17	−11
Outpatient appointments	115	0.90	67	0.52	−48
Online/telephone Outpatient appointments	55	0.43	33	0.26	−22
Day case appointments	13	0.10	12	0.09	−1
Hospital Inpatient days	45	0.35	5	0.04	−40
Hospital Inpatient episodes	9	0.07	3	0.02	−6
General practitioner appointments	194	1.52	139	1.09	−55
General practitioner telephone/online appointments	110	0.86	76	0.59	−34
General practitioner home visits	6	0.05	4	0.03	−2
Appointments with other healthcare professionals (face‐to‐face)	194	1.52	96	0.75	−98
Appointments with other healthcare professionals (telephone/online)	52	0.41	21	0.16	−31
Appointments with other healthcare professionals (home visits)	9	0.07	7	0.05	−2
N	128		128		

Cost analysis based on published NHS unit costs (see Supporting Information S2) showed an estimated difference in self‐reported healthcare costs of £36,821 over the 12 weeks, equivalent to £288 per patient (Table 3). As inpatient episodes and day case appointments were infrequent but carried higher unit costs, sensitivity analyses were undertaken. When inpatient episodes were excluded, the estimated cost difference was £23,789, equivalent to £186 per participant. When both inpatient episodes and day case appointments were excluded, the estimated cost difference was £23,442, equivalent to £183 per participant. These estimates should be interpreted cautiously because all healthcare use was self‐reported, the analysis was uncontrolled, and the evaluation was not designed as a formal cost‐effectiveness analysis.

**TABLE 3:** Estimated differences in self‐reported healthcare costs over the 12 weeks (complete responders).

	Difference in healthcare usage (Follow‐up minus baseline)	Total estimated cost impact over period (12 weeks), £
Healthcare service		
Accident and Emergency visits	−11	−3003
Outpatient appointments	−48	−9792
Online/telephone Outpatient appointments	−22	−4488
Day case appointments	−1	−347
Hospital Inpatient episodes	−6	−13,032
General practitioner appointments	−55	−2695
General practitioner telephone/online appointments	−34	−689
General practitioner home visits	−2	−588
Appointments with other healthcare professionals (face‐to‐face)	−98	−1588
Appointments with other healthcare professionals (telephone/online)	−31	−502
Appointments with other healthcare professionals (home visits)	−2	−97
		−36,821

*Note*: Other professionals assumed to be a nurse/podiatrist.

### 3.3 Qualitative Findings

Qualitative interviews conducted with a purposive sample of participants (*n* = 26) provided important context to the quantitative findings and helped to illuminate the mechanisms through which the Diabetic Wellness Kit supported self‐management. Five key themes were identified through thematic analysis: (1) increased awareness and routine foot care, (2) practical assistance with daily tasks and mobility, (3) positive lifestyle and wellbeing impact, (4) perceptions of usefulness, redundancy, and preferences, and (5) enhanced confidence and independence. Participants frequently reported that the kit helped to establish or reinforce daily foot care routines, including moisturising and visual inspection of the feet. These activities were described as simple but effective in improving foot condition and reducing anxiety about complications. Several tools were highlighted as both practical and easy to integrate into daily life. For some individuals, the kit also acted as a prompt for broader lifestyle changes, such as increased physical activity and weight management.

The intervention appeared to strengthen participants' sense of confidence and autonomy, reflecting core principles of self‐management. Users described feeling more capable of managing their health independently, and many expressed a willingness to recommend the kit to others or purchase it themselves. At the same time, some respondents noted areas for improvement, including the desire for greater personalisation of kit contents and the importance of ensuring product quality. Although access to digital educational materials was included as part of the intervention, usage of the online resources was not formally tracked. However, most interviewees who were asked about the website reported being aware of its content and found it helpful in supporting their ongoing self‐care. A full account of the qualitative methodology, coding process, and illustrative quotations is available in the Supplementary Material.

## 4 Discussion

Globally, type 2 diabetes mellitus presents a growing challenge to healthcare systems, especially in socioeconomically deprived areas where complications are more common and access to preventative care is lower. This evaluation was designed to explore early changes in wellbeing, routines and self‐management following real‐world use of the kit, rather than to test clinical effectiveness. As noted in this evaluation, engagement with structured education and routine foot screening remains suboptimal. Interventions that are accessible, affordable and capable of promoting daily self‐care are urgently needed [21].

The Diabetic Wellness Kit was developed to support such needs, combining practical assistive tools with educational materials in a low‐cost format. This evaluation indicates that the intervention was positively received and associated with improvements in self‐efficacy, health‐related quality of life, and a reduction in self‐reported healthcare utilisation. The findings contribute to a growing body of evidence supporting the use of simple, structured interventions to enhance self‐management in chronic disease. Our quantitative findings indicate an increase in EQ 5D utility score over a 12‐week period, with utility scores increasing from 0.61 to 0.70 and 65% of complete responders reporting improvement.

In parallel, there was a reduction in healthcare resource use across all major service categories, with an estimated average cost difference of £288 per patient. These findings are consistent with previous evidence that self‐management support and personalised care planning for long‐term conditions can improve health outcomes, self‐management capability, and, in some settings, healthcare use and costs [8, 22, 23]. The short‐term changes observed in this evaluation may represent early signals of improved engagement with self‐care, but they do not indicate long‐term clinical outcomes and should be interpreted with appropriate caution. This is particularly important because all data were self‐reported and subject to recall bias. Despite instructions to report only diabetes‐related resource use, misclassification is possible. Because inpatient and day‐case services carry disproportionately high unit costs, their low frequency and potential misclassification may have influenced overall cost estimates. Nonetheless, even when inpatient episodes were excluded, we observed a cost difference of £186 per patient and reductions across every other service category. This consistent reduction across all categories suggests possible changes in reported service use. When both inpatient episodes and day case appointments were excluded, the estimated cost difference was £183 per participant. As programme administration costs were not included, these findings should not be directly interpreted as cashable savings at the system level.

Only complete responders were included in the main analysis. While no formal missing data analysis was conducted, baseline‐only responders and complete responders had broadly similar quality of life scores, sex distribution and BMI. However, complete responders were older on average, and the high level of attrition means that responder bias is likely. Participants who completed follow‐up may have been more motivated, more able to engage with the kit, more confident completing digital questionnaires, or less unwell than those who did not complete follow‐up. Conversely, participants with worsening health, greater healthcare use, or lower engagement may have been less likely to respond. The likely direction of this bias is therefore towards overestimating positive changes in quality of life, self‐management, and healthcare use among the wider group of kit recipients. This limits generalisability. The observed differences in healthcare utilisation may also reflect factors other than kit use, including routine scheduling of appointments, regression to the mean, seasonal variation, or concurrent changes in health status and treatment. While our findings are promising, future studies should aim to triangulate self‐report data with clinical records and include longer follow‐up to explore sustained impact. Qualitative data provided insight into how the kit supported self‐management. Participants frequently reported forming new routines around foot care, such as daily use of moisturiser and inspection tools, with improvements in skin condition and confidence. Tools such as the shoe horn and insoles were cited as improving independence and mobility. A smaller group reported broader lifestyle changes, including increased physical activity and weight loss. These findings are consistent with behavioural models that emphasise the importance of autonomy, competence, and integration into daily routines. The kit did not rely on formal education alone; rather, it provided tangible tools that enabled immediate and visible action, enhancing the perceived relevance and usability of self‐care advice. Some participants expressed preferences for a more tailored kit, noting that certain items (e.g., pedometers, tape measures) were less useful to them or redundant due to existing devices. Others suggested adding tools for circulation or nail care. Bringing together the quantitative and qualitative findings helped to show not only what changes were reported, but also how participants described integrating the kit into daily routines.

Although the kit was standardised for evaluation purposes, these findings highlight the potential value of future personalisation, particularly for people with co‐ or multi‐morbidity. An automated screening and decision support tool could support this process. The intervention was generally viewed as affordable and high value. The longevity of kit components was not assessed, and further study is needed to examine longer‐term cost implications.

### 4.1 Limitations

This service evaluation has several important limitations that should be considered when interpreting the findings. The study used a simple before and after design without a comparison group, which means that no causal conclusions can be drawn. Any changes seen over the 12‐week period may have been influenced by natural variation, seasonal effects, regression to the mean, or wider changes in access to care rather than the kit itself.

Follow‐up was completed by only a small proportion of those who received a kit. Although complete responders and baseline‐only responders had broadly similar baseline utility scores and healthcare resource use, the risk of responder bias remains high. People who chose to complete follow‐up may have been more motivated, more engaged, or more able to use the kit, which could overstate positive findings.

All outcome measures were based on self‐report. This includes quality of life, self‐management experiences, and healthcare use. Self‐reported information is subject to recall errors and may not fully match routine clinical records. This is especially relevant for healthcare use and cost estimates, where small errors in recall can lead to large differences in calculated costs. The economic findings should therefore be viewed as exploratory and not as confirmed cost changes.

Clinical variables were not extracted from patient records. We therefore did not have data on HbA1c, duration of diabetes, comorbidities, diabetes treatment, medication changes, neuropathy status, previous ulceration, or baseline foot risk. This limits interpretation and means that we cannot assess whether outcomes differed by clinical risk group or diabetes severity.

The follow‐up period was short. Twelve weeks is suitable for exploring early changes in routines, confidence and wellbeing, but it is not long enough to assess effects on long‐term diabetes outcomes, foot complications, or sustained healthcare use. The evaluation also did not include a formal fidelity assessment or continuous monitoring of individual kit component use, which limits interpretation of which elements of the intervention were most influential. Longer follow‐up using routine data would be needed to explore these outcomes.

## 5 Conclusion and Recommendations

This evaluation identified improvements in health‐related quality of life and differences in self‐reported healthcare utilisation among participants who received the Diabetic Wellness Kit. The intervention was positively received and appears to offer a practical approach to supporting daily self‐care. Further controlled studies with longer follow‐up are needed to determine whether these observations are sustained and attributable to the intervention. Future implementation and research should focus on:

- Targeted rollout in high‐need populations, particularly where structured education and preventative care are underused.
- Development of a personalisation mechanism, such as an automated decision‐support tool, to match kit contents to individual needs.
- Integration with existing diabetes services as a complementary intervention.
- Longitudinal evaluation to assess durability of impact, potential effects on clinical outcomes, and health economic value over time.

If refined and appropriately targeted, such low‐cost interventions may provide a practical way to support self‐management for people living with T2DM, alongside usual diabetes care.

## Author Contributions

**Nitish Nachiappan:** conceptualisation, methodology, writing – original draft, writing – review and editing; **David A. Scott:** methodology, formal analysis, writing – review and editing; **Mark Stone:** resources, writing – resources, review and editing; **Nachiappan Chockalingam:** conceptualisation, methodology, formal analysis, investigation, resources, data curation, funding acquisition, writing – original draft.

## Ethics Statement

This project fully adhered to the Declaration of Helsinki. It was conducted as a service evaluation of an existing NHS supported intervention and was reviewed using the Health Research Authority, UK decision tool (https://www.hra-decisiontools.org.uk/research/), which confirmed that NHS research ethics approval was not required. No clinical procedures or changes to treatment were introduced as part of this evaluation. All participants provided written informed consent before taking part in the questionnaire and interview components. Data were collected and stored in accordance with NHS information governance requirements.

## Conflicts of Interest

The authors declare the following financial interests/personal relationships which may be considered as potential competing interests. NC and NN are currently directors of Alegre limited, which owns and plans to commercially offer MyHealthKits. None of the other authors have declared any competing interests. NC was the primary contact for the funding agency and this project was a part of a portfolio of projects funded through HTAAF initiative.

## Supporting information


Supporting File 1

Supporting File 2

## Data Availability

The data that support the findings of this study are available on request from the corresponding author. The data are not publicly available due to privacy or ethical restrictions.
