# Supplementary material for: Evaluating a Structured Self‐Management Kit for Type 2 Diabetes: A Mixed Methods Service Evaluation in Primary Care
Source: J Eval Clin Pract. 2026 Nov 4;32(8):e70724. doi: 10.1111/jep.70724 (PMC13734456; doi:10.1111/jep.70724)
Supplement: Supplementary file 1 [file JEP-32-0-s001.docx]

**Supplementary Material 1: Qualitative Findings**

***Theme 1: Increased Awareness and Routine Foot Care***

The most frequently reported benefit of the Diabetic Wellness Kit was its role in promoting regular and proactive foot care. Participants described developing daily habits around foot moisturising, checking for problems, and maintaining overall skin condition. The foot cream, telescopic inspection mirror, foot file, and socks were consistently described as the most useful items.

Many participants commented that they had not previously checked the soles of their feet regularly, but the mirror allowed them to do so with greater ease and confidence. One person shared, *“The foot mirror is the best as it helps me self-check my feet… I found it so very helpful.”* Another stated, *“I am starting to look after my feet better.”* The moisturiser was widely praised, with one participant noting, *“Cream has worked very well on the dry skin on my feet; I noticed a difference as fast as the next day of using it.”*

This shift in self care was linked to a new sense of routine and purpose. One participant shared, *“The file and cream have improved my foot health. My heels don’t crack, and they’re much softer now.”* These small, repeated behaviours appeared to contribute to sustained improvements in foot condition and self-efficacy. These behavioural changes often served as a foundation for increased confidence and self-reliance, explored further in Theme 5.

***Theme 2: Practical Assistance with Daily Tasks and Mobility***

Several participants commented on how individual components of the kit helped them manage daily physical tasks more independently. The long-handled shoe horn, in particular, was mentioned as reducing the need to bend down, which was important for those with balance or joint concerns. As one user put it, *“The metal shoe horn is better than my old plastic one because I don’t have to bend down and go dizzy.”*

The insoles and socks were also recognised as improving comfort during walking and standing. One participant explained, *“The insoles and socks have made my feet a lot more comfortable on a daily basis and encouraged me to focus on foot comfort.”* The socks were valued not only for comfort but also for fit and circulation support: *“The socks are extremely comfortable and don’t leave indentations on my legs.”*

***Theme 3: Positive Lifestyle and Wellbeing Impact***

For a smaller number of participants, the use of the kit appeared to act as a motivator for broader changes in self care and health management. These included increased physical activity, improved weight control, and more active monitoring of diabetes-related health indicators. One participant said, *“My sugars are lower, and I lost 4 kg since I started paying more attention to my health with the kit.”* Another shared, *“I use the tape measure each month and the file helps keep my feet in good condition—thank you.”*

Some participants linked the pedometer to increased walking and physical activity. For example, *“I’ve recently started using the pedometer when I walk to work.”* Though not universally relevant, the pedometer supported behaviour change for those already considering improvements to activity levels.

***Theme 4: Perceptions of Usefulness, Redundancy, and Preferences***

While overall feedback was positive, a number of participants reflected on personal needs and preferences that influenced how useful they found particular items. Some participants suggested the kit could be improved by allowing customisation or choosing items from a list. As one person put it, *“I would prefer to choose from a list.”*

There were occasional mentions of redundancy, especially when users already owned a pedometer or used a smartwatch to track steps. A few individuals noted that some items, such as large mirrors or tape measures, may be more appropriate for people with more advanced diabetes or specific mobility needs. Others suggested that additional items, like nail care tools or circulation aids, could enhance the usefulness of the kit. Two isolated concerns were raised regarding the physical quality of some items. One recipient reported issues with the durability of two components, which were subsequently checked and replaced. Another participant mentioned being unable to tolerate the scent of the bamboo socks, although this was not substantiated upon further investigation and no similar complaints were received. These instances highlight the importance of ongoing quality assurance and the potential value of offering alternative product options where appropriate.

***Theme 5: Confidence, Independence, and Recommendation***

Many participants expressed a boost in confidence following use of the kit. Being able to manage their own foot care without relying on others was a recurring theme. Participants also commonly reported recommending the kit to others or expressing a willingness to do so. Several noted that they had either bought similar products for family members or would be happy to pay a modest amount (commonly around £40) for the kit if made available commercially.

One participant neatly summarised this empowerment: *“Now I care more for my feet.”* Others described the benefits in relation to independence, motivation, and encouragement to maintain good habits.

Although all participants were provided with access to a dedicated website containing lifestyle advice, diabetic-friendly recipes, and instructional videos, no formal monitoring of access or usage statistics was conducted as part of this evaluation. However, during qualitative interviews, individuals who were asked about the website indicated that they were aware of its contents and found it relevant to their self-management needs. The extent to which this digital resource supported behaviour change remains unclear, and its utility should be explored in more detail in future evaluations.
