# Supplementary material for: Evaluating a Structured Self‐Management Kit for Type 2 Diabetes: A Mixed Methods Service Evaluation in Primary Care
Source: J Eval Clin Pract. 2026 Nov 4;32(8):e70724. doi: 10.1111/jep.70724 (PMC13734456; doi:10.1111/jep.70724)
Supplement: Supplementary file 2 [file JEP-32-0-s002.docx]

**Supplementary Material 2: Assumed Costs**
